# High-Resolution Phenotypic Profiling Defines Genes Essential for Mycobacterial Growth and Cholesterol Catabolism

**DOI:** 10.1371/journal.ppat.1002251

**Published:** 2011-09-29

**Authors:** Jennifer E. Griffin, Jeffrey D. Gawronski, Michael A. DeJesus, Thomas R. Ioerger, Brian J. Akerley, Christopher M. Sassetti

**Affiliations:** 1 Department of Microbiology and Physiological Systems, University of Massachusetts Medical School, Worcester, Massachusetts, United States of America; 2 Department of Computer Science, Texas A&M University, College Station, Texas, United States of America; 3 Howard Hughes Medical Institute, Chevy Chase, Maryland, United States of America; University of Washington, United States of America

## Abstract

The pathways that comprise cellular metabolism are highly interconnected, and alterations in individual enzymes can have far-reaching effects. As a result, global profiling methods that measure gene expression are of limited value in predicting how the loss of an individual function will affect the cell. In this work, we employed a new method of global phenotypic profiling to directly define the genes required for the growth of *Mycobacterium tuberculosis*. A combination of high-density mutagenesis and deep-sequencing was used to characterize the composition of complex mutant libraries exposed to different conditions. This allowed the unambiguous identification of the genes that are essential for Mtb to grow *in vitro,* and proved to be a significant improvement over previous approaches. To further explore functions that are required for persistence in the host, we defined the pathways necessary for the utilization of cholesterol, a critical carbon source during infection. Few of the genes we identified had previously been implicated in this adaptation by transcriptional profiling, and only a fraction were encoded in the chromosomal region known to encode sterol catabolic functions. These genes comprise an unexpectedly large percentage of those previously shown to be required for bacterial growth in mouse tissue. Thus, this single nutritional change accounts for a significant fraction of the adaption to the host. This work provides the most comprehensive genetic characterization of a sterol catabolic pathway to date, suggests putative roles for uncharacterized virulence genes, and precisely maps genes encoding potential drug targets.

## Author Summary

The adaptation of a bacterial pathogen to the environments encountered during infection requires the wholesale remodeling of cellular physiology. One of the major changes encountered upon infection is nutritional, as the bacterium is forced to utilize compounds scavenged from the host. *Mycobacterium tuberculosis* has the unusual ability to use host cholesterol as a source of carbon and energy, and this capacity is required for persistence in animal models. However, our ignorance of the biochemical pathways involved in sterol degradation has limited our ability to assess the importance of this carbon shift in shaping the metabolic state of the bacterium. In this work, we developed a new method to quantitatively profile the fitness of thousands of mutants simultaneously. This allowed the identification of each bacterial gene that is required for the bacterium to grow using cholesterol as a carbon source. Reconstruction of the pathways comprised by these essential gene products revealed that adaptation to cholesterol required widespread metabolic changes. These genes account for a significant fraction of the bacterial functions important for growth in animal tissues, suggesting that the physiology of this intracellular pathogen is shaped by carbon sources available in this environment.

## Introduction

*Mycobacterium tuberculosis* (Mtb) is a deadly human pathogen, which is estimated to infect one third of the world's population and killed 1.7 million people in 2009 alone [1]. This bacterium must adapt to a number of different microenvironments over the course of a chronic infection [2], and understanding the physiological state of the pathogen in these sites is central to the design of more effective antitubercular drugs. Defining these metabolic adaptations requires a holistic understanding of the cellular pathways that are critical for survival in each specific environment. While whole-genome methods to profile mRNA, protein, or transcription factor binding are valuable for understanding cellular responses, gene regulation has proven to be a poor predictor of essentiality [3], [4]. Therefore, genome-wide approaches to accurately and directly assess the relative contribution of each gene to the growth and survival of *M. tuberculosis* are needed.

The genome-scale methods initially used to predict gene essentiality in bacteria employed either microarray hybridization [5], [6] or conventional sequencing [7], [8], [9] to map the sites of transposon insertions in large libraries of random mutants and identify regions of the chromosome that were unable to sustain mutation. Both of these methods suffer from significant limitations. Microarrays are not able to precisely map insertions, resulting in ambiguity regarding the precise location of an essential region. While conventional sequencing can surmount this problem, it is both cumbersome and expensive to apply this method to the very complex libraries necessary for the unambiguous identification of essential genes. Finally, neither of these approaches can be used to quantitatively compare the compositions of mutant pools over more than a very small dynamic range.

In order to precisely define the genes that are important for the growth of Mtb, we used highly parallel Illumina sequencing to characterize transposon libraries. This approach achieves single base pair resolution of insertion sites in very complex libraries, resulting in the precise identification of essential open reading frames. Furthermore, the depth of sequencing that is possible allowed the relative abundance of individual mutants to be accurately assessed in libraries grown under different conditions. Using the latter approach, we comprehensively defined the pathways required for the bacterium to grow on cholesterol, a critical nutrient during infection. These previously ill-defined pathways account for an unexpectedly large fraction of the genes required for survival in animal models of TB, providing new functional insight into the adaptation to the host environment.

## Results

### Transposon mapping and identification of genes required for *Mtb* growth *in vitro*

A library of transposon mutants consisting of approximately 10^5^ independent insertion events was generated in the H37Rv strain of Mtb using a modified *himar1* based transposon [5]. Replicate portions of this library were grown for 12 generations in defined media containing different carbon sources, and chromosomal DNA was extracted from each pool. This DNA was randomly fragmented, ligated to asymmetric adapters, and transposon chromosome junctions were amplified using PCR. Illumina sequencing was then used to determine the sequences of 6 to 8 million transposon:chromosome junctions per library. 95–99% of these sequences represented the specific amplification of transposon insertions (see methods) and were used for subsequent analyses.

**Figure 1:**
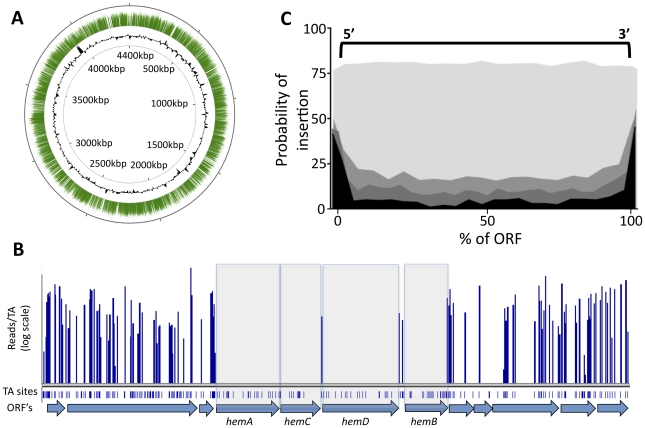
Transposon insertions are randomly distributed, and gaps in transposon coverage correspond to predicted essential ORFs. (A) Transposon:chromosome junctions from high density mutant libraries were identified by deep sequencing. The number of sequence reads corresponding to each insertion site is represented as green bars mapped onto the circular chromosome of *M. tuberculosis* H37Rv. Black contour represents the GC content of the chromosome (G+C content greater or less than 50% are represented as contours outside or inside the ring, respectively). Nucleotide positions are indicated (kilobases). (B) Essential heme biosynthetic genes are devoid of insertions. The number of sequence reads (“reads/TA”) is shown for the indicated region of the H37Rv chromosome. Potential TA dinucleotide insertions sites are indicated. (C) Each potential TA insertion site in the genome was assigned a position relative to the nearest ORF. The probability of detecting an insertion at each ORF position (per 20 TA window) is plotted on the y-axis. Genes predicted to be nonessential (p value >0.5, shaded lightest gray) show no position-dependent insertional bias. In contrast, genes predicted to be required for growth with different degrees of confidence (p<0.2, p<0.01, p<0.00005, indicated in progressively darker shading) show a strong bias against insertions in the open reading frame.

The transposon used in these studies requires a TA dinucleotide insertion site. We identified transposon insertions at 44,350 of the 74,605 possible TA sites in the genome (Table S1). This corresponded to approximately one insertion every 100 base pairs on average and was consistent with the previously described random insertion of this element [9], [10], [11]. The identified insertions were distributed throughout the chromosome (Figure 1A), with the exception of small gaps, many of which corresponded to known essential genes in which we expected insertions to be lethal. For example, a small region of the chromosome encodes several of the enzymes required for the synthesis of the essential cofactor, heme, (Figure 1B) and we found that gaps in transposon coverage precisely mapped to these open reading frames (ORFs).

In contrast, we found that other likely essential genes could harbor a small number of insertions (not shown), consistent with previous observations [12]. Therefore, we globally defined essential ORFs by searching for genes with statistically significant gaps in transposon insertion coverage, instead of simply compiling those that were absolutely devoid of insertions. We identified the longest sequence of TA sites lacking insertions within a given gene, and determined the likelihood of a non-essential gene harboring a gap of that length (modeled as a Bernoulli process) as a function of the total number of TA sites in the ORF. We then calculated the probability of the longest observed gap arising by chance, relative to the Extreme Value Distribution (Table S2). This analysis method exploited the value of the high-resolution data provided by deep sequencing, while allowing for permissive insertion sites in essential genes.

While the *himar1* transposon used in these studies has been shown to integrate relatively randomly, it remained possible that some regions of the chromosome were resistant to transposition and genes in these regions would erroneously appear to be required for viability. To address this possibility we determined if the identified gaps in transposon coverage corresponded to protein-coding regions. As shown in Figure 1C, the probability of identifying a transposon insertion in or near a putative essential region is highly correlated with the position of the corresponding predicted ORFs. This probability increased at the extreme ends of the gene, likely because many insertions at these sites do not disrupt gene function. Together, these data indicate that regions devoid of sequenced transposon insertions represent protein-coding genes that are important for the growth or survival of the organism and not regions that are less accessible to transposition. Not surprisingly, we were also able to identify significant gaps in transposon coverage that did not correspond to predicted ORFs. Determining which of these regions correspond to unannotated protein-coding genes, essential untranslated RNAs, or regulatory motifs will require further study.

### Comparison with previous studies

**Figure 2:**
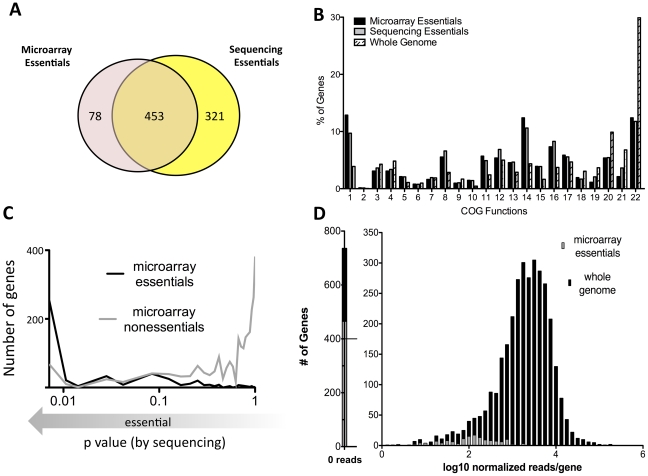
Defining essential genes with deep sequencing is more sensitive and precise than previous approaches. (A) Gene sets predicted to be essential via microarray [6] are compared with genes predicted to be essential via deep sequencing.(p<0.05) (B) Microarray and deep sequencing identify essential genes sets consisting of similar predicted functions, which differ from the genome as a whole. Clusters of Orthologous Groups (COG) categories associated with essential genes predicted by microarray or deep sequencing (p<0.05) are plotted along with the predictions for the entire genome. COG functions are: 1) Translation, ribosomal structure, biogenesis, 2) RNA processing, modification, 3) Transcription, 4) Replication, recombination, repair, 5) Cell cycle control, cell division, chromosome partitioning, 6) Defense mechanisms, 7) Signal transduction mechanisms, 8) Cell wall/membrane/envelope biogenesis, 9) Cell motility, 10) Intracellular trafficking, secretion, vesicular transport, 11) Posttranslational modification, protein turnover, chaperones, 12) Energy production, conversion, 13) Carbohydrate transport, metabolism, 14) Amino acid transport, metabolism, 15) Nucleotide transport, metabolism, 16) Coenzyme transport, metabolism, 17) Lipid transport, metabolism, 18) Inorganic ion transport, metabolism, 19) Secondary metabolites biosynthesis, transport, catabolism, 20) General function prediction only, 21) Function unknown, 22) No assignment. (C) Microarray predictions are generally, but not absolutely, consistent with deep sequencing. The probability of essentiality defined via deep sequencing is plotted on the x-axis (as log p value) for genes previously predicted by microarray to be either essential (black) or nonessential (gray). (D) Mutations in nonessential genes previously predicted to be required for growth cause quantitative fitness defects. Normalized sequence reads per gene (log scale) are plotted for all genes (black) and genes predicted to be essential by microarray (gray). Previously defined essential genes with detectable insertions are associated with low sequence read counts, suggesting quantitative underrepresentation in the library. Genes containing fewer than seven TA's were excluded from all analyses, since statistically-significant predictions could not be made.

This deep sequencing-based approach for identifying growth-attenuated mutants both validates and improves upon our previous microarray-based studies. About 15% of the Mtb genome, or 614 genes, were previously predicted to be required for optimal growth *in vitro* using microarray hybridization to map insertions sites [6]. Our current deep sequencing approach identified a somewhat larger number of genes, 774, that contain statistically-significant gaps in coverage (p<0.05). These gene sets largely overlap and encode a similar distribution of cellular functions, which are quite different from the genome as a whole (Figure 2A, B).

Despite the gross similarities between the two gene sets, we also found differences (Figure 2A, C). Some of these differences were attributable to minor alterations in growth conditions. The current study used a more defined media than the standard 7H10 agar employed previously. However, it is likely that most discrepancies are due to the increased resolution and dynamic range of the current method. For example, approximately one half of the genes that deep sequencing identified as essential (p<.05) could sustain a small number of insertions (Table S2). The presence of these permissive insertion sites likely caused many of these genes to be deemed nonessential by the previous lower resolution method (Figure 1C).

The second major difference between these datasets is a result of the sequencing depth that was employed. Because of the limited dynamic range of the microarray method (typically 3–10 fold), it was difficult to differentiate between mutants that were truly nonviable and those that were merely significantly underrepresented. The increased dynamic range of deep sequencing-based mapping allowed for this differentiation. Indeed, the insertions we identified in genes previously predicted to be essential were associated with a significantly lower number of sequence reads than the genome as a whole (Figure 2D). This suggested that these mutants suffered from a fitness defect but remained viable. Based on the average number of sequence reads we detected in nonessential ORFs (173 reads/ORF), we estimate that genes containing statistically significant gaps in coverage correspond to mutants that are more than 100 fold underrepresented in the pool and are therefore essentially nonviable.

### Genes required for growth on cholesterol

This method provided a quantifiable assessment of transposon library composition, indicating that the comparison of mutant pools selected under different conditions should be possible. We compared pools grown in glycerol, a standard *in vitro* carbon source, with pools grown in cholesterol, a critical carbon source during infection [13], [14], [15], in order to define the genes and pathways required for the catabolism of cholesterol.

**Figure 3:**
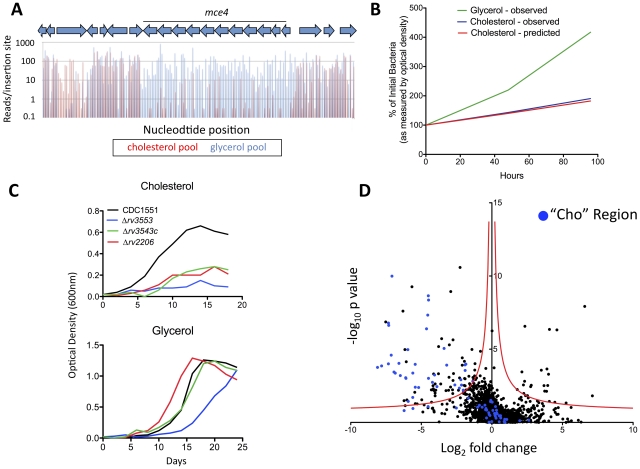
Genes required for growth on cholesterol. (A) Mce4 mutants are specifically underrepresented in the cholesterol-grown pool. Transposon libraries grown for 12 generations in media with either glycerol or cholesterol as a primary carbon source were compared by deep sequencing. Normalized sequence reads for individual insertions sites throughout the Mce4 operon are shown following growth in glycerol (blue) and cholesterol (red). The average underrepresentation of Mce4 mutants in the cholesterol-grown pool corresponds to a predicted 31% growth disadvantage per generation. (B) The number of sequence reads per TA provides an accurate estimate of relative growth rates. The experimentally determined growth curves of a Mce4 deletion mutant in the indicated media are compared to the growth rate of Mce4 mutants predicted in panel A. Log phase growth is plotted as percentage of initial bacterial number. (C) Mutants predicted to be required for cholesterol utilization display the predicted phenotypes. Transposon mutants were grown in minimal media with the indicated primary carbon sources and growth was monitored by optical density. This experiment was repeated three times with similar results. (D) Identification of genes that are differentially required for growth. For each gene, the ratio of normalized sequence reads per insertion site (cholesterol pool/glycerol pool) is plotted on the x-axis (“fold change”). Y-axis represents the significance of each of these changes in representation (p value). A hyperbolic function was used to define genes that were differentially represented. The asymptotes of these curves are 0 for the log_2_ fold change and −0.07 for the log_10_ p value. Genes containing fewer than two TA sites were excluded from the analysis. Genes in blue represent those within the predicted cholesterol region.

In principle, the number of sequence reads associated with a specific insertion should be proportional to the relative abundance of the corresponding mutant in the library. We predicted that the sequencing depth used in our study would allow the relative abundance of each mutant in different libraries to be compared over a 100–1000 fold range. To verify this, we specifically analyzed the genes encoding the previously characterized cholesterol uptake system encoded by the ten-gene *mce4* operon. Disruption of Mce4 function by deleting an essential transmembrane component has been shown to cause specific defects in cholesterol uptake and growth in this carbon source [14]. Consistent with these observations, we found that mutations in every gene of this operon, as well as the associated ATPase [16], resulted in severely impaired growth in cholesterol relative to glycerol (Figure 3A). The degree of this selective underrepresentation predicted that these mutants suffered a 31% growth disadvantage per generation, which is very similar to the experimentally observed doubling time of an isolated Mce4 mutant in these media (Figure 3B). The predicted cholesterol-specific growth defects of three additional transposon were experimentally verified (Figure 3C), further validating the method. We concluded that the number of sequence reads associated with a given mutant could be used to accurately predict its growth rate.

Using this metric as an estimate of relative abundance, we compared the genome-wide data sets. As expected, we found that the representation of most mutants was similar in both pools (Figure 3D). Due to the pre-selection of the library on complex glycerol-containing media before passage in single carbon sources, relatively few mutants were found to be specifically required for growth in glycerol. In contrast, a much larger number of mutants were underrepresented in the cholesterol-grown pool. To define differentially represented mutants, we employed a cutoff using a continuous function that equally weighted statistical significance and the magnitude of the change in representation (Figure 3D). To reduce the potential impact of biases introduced during PCR amplification and sequencing, statistical significance was calculated using each individual insertional mutant in the replicate libraries as an independent data point. Ninety-six genes met these statistical criteria and were therefore predicted to be important for growth on cholesterol (Table S3 and 4).

While the cholesterol catabolic pathway of Mtb has only been partially defined, all of the known and predicted catabolic enzymes are encoded in a distinct ∼50 kb region. This “Cho region” comprises over 80 genes and includes the *mce4* operon [15], [17]. While the genes we found to be required for growth in cholesterol were enriched in this region, the majority (>60%) were distributed elsewhere in the chromosome (Figure 3D). These genes encoded a wide variety of predicted enzymatic activities that could be categorized into the following three functional groups associated with cholesterol utilization.

### Sterol ring degradation

**Figure 4:**
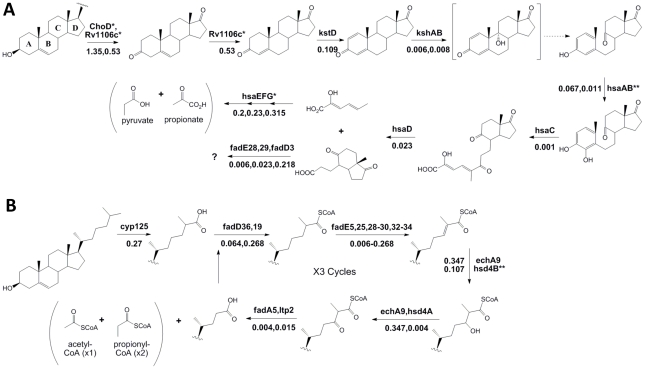
Phenotypic profiling predicts the cholesterol catabolic pathway. Proposed chemical transformations necessary for cholesterol degradation are divided into sterol ring cleavage and opening (A) and side-chain degradation (B). Enzymes known to or predicted to function at each step are noted with their respective fold change (sequence reads in cholesterol/sequence reads in glycerol) below. Dotted lines represent spontaneous reactions. We did not find enzymes with marked * to be significantly required for growth on cholesterol. Enzymes marked with ** indicate those where we are unable to report significance due to insufficient data. The specific β-oxidation steps mediated by fadE28 and fadE29 are difficult to predict based on their homology to testosterone degrading enzymes and therefore, we have assigned them to both equally likely steps.

Following import, cholesterol is degraded via β-oxidation of the side-chain, and ring cleavage to open the steroid nucleus (Figure 4). Our phenotypic data supports and augments the current predicted pathway for sterol ring degradation. The first step in cholesterol catabolism requires the oxidation of the 3β-hydroxyl group and isomerization of the resulting cholest-5-en-3-one to cholest-4-en-3-one. Two distinct enzymes have been suggested to be involved in this transformation; a ketosteroid dehydrogenase, Rv1106c, and a cholesterol oxidase, ChoD [18], [19]. We found that only mutations in the ketosteroid dehydrogenase gene caused a significant growth defect, whereas *choD* mutants appeared to grow at a similar rate in both carbon sources (Figure 4A and Table S3).

While all of the subsequent predicted steps of sterol ring degradation were critical for growth in cholesterol, the degree of importance varied depending on the position of the enzyme in the pathway. We found that *hsaEFG* mutants, which are predicted to be unable to further degrade 2-hydroxyhexa-2,4-dieneoic acid (HHD) to propionate and pyruvate [15], were only modestly defective in cholesterol media, in contrast to the drastic growth attenuation of mutants lacking functions at earlier steps. This is likely because HsaEFG act distal to a branchpoint in the pathway and the mutant bacteria could grow, albeit slowly, by fully catabolizing the portion of the molecule containing rings C and D.

### Side-chain degradation

In the related actinomycete, *Rhodococcus jostii* RHA1, the initial hydroxylation of C26 during side-chain degradation is mediated by the cytochrome P450 encoded by the *cyp125* gene [20]. In Mtb, the Cyp125 ortholog serves a similar function [21], [22], although Cyp142 has been shown to serve a functionally redundant role [23]. Here, we identify Cyp125 as the sole monooxygenase significantly required for growth on cholesterol (Table S3 and S4). However, the modest 5.5 fold underrepresentation of *cyp125* mutants in cholesterol supports the previously observed redundancy between cytochrome P450 enzymes.

Many enzymes resembling those predicted to be required for the β-oxidation of the cholesterol side-chain are encoded in the Cho region. Surprisingly, we found significant functional clustering even within this region. None of the predicted β-oxidation genes encoded within the first half of the Cho region were required for growth on cholesterol, except *hsd4A* (Table S3). Instead, we found that almost all of those encoded in the second half were required (including *ltp2, fadE29, fadE28, fadA5, fadE30, FadE32, fadE33, fadE34,* and *hsd4B)*. Notably, we also identified predicted β-oxidation genes encoded outside of the Cho region that had not yet been implicated in cholesterol degradation (including *fadE5, echA9, fadD36,* and *fadE25*) (Figure 4B). Together, these genes could perform the three rounds of β-oxidation predicted to be required for full catabolism of the C17 side-chain, although we cannot exclude the participation of other functionally-redundant enzymes, or the possibility that some of these genes participate in degradation of the steroid nucleus.

### Intermediary metabolism

Cholesterol catabolism is thought to result in the production of a mixture of metabolites including the C2 unit acetyl CoA, the C3 unit propionyl CoA, and pyruvate [14], [15], [24]. The incorporation of acetyl- and propionyl-CoA into the TCA cycle of Mtb likely requires the coordinated activity of the glyoxylate- and methylcitrate-cycles. The *icl* gene of *M. tuberculosis* H37Rv encodes a multifunctional protein with both isocitrate lyase and methylisocitrate lyase activity, which is required for both pathways [25], [26]. We found *icl* mutants to be 125-fold underrepresented in the cholesterol-grown pool (Table S3), verifying an important functional role. In addition, our data suggests that the expression of this multifunctional enzyme may be rate limiting during growth on cholesterol, as the mutation of *RamB*, a negative regulator of *icl* expression [27], resulted in the opposite effect (a nine-fold overrepresentation in the cholesterol-grown pool).

Growth on lipids as a primary carbon source requires gluconeogenesis, and different metabolites can be used to fuel this pathway. Following growth on cholesterol, we found that *ppdK* mutants were severely underrepresented (Table S3). This gene encodes pyruvate phosphate dikinase, which can mediate the conversion of pyruvate to phosphoenolpyruvate (PEP), the first committed step of gluconeogenesis. Together, these observations define pathways required for the assimilation of the three metabolites that most likely result from cholesterol catabolism.

## Discussion

In this work, we have significantly refined our understanding of the cellular functions necessary for the viability of Mtb. While others have used similar deep sequencing methods to map insertion sites in other organisms [28], [29], [30], [31], [32] our new analytical tools allowed the statistically rigorous prediction of the genes essential for the viability of Mtb. The majority of these essential genes are consistent with those found by previous microarray-based methods. However, significant differences in these predictions were also noted, and the majority of these are attributable to technical and analytical refinements. As a result, this work provides a much more precise and statistically rigorous global assessment of essentiality than previously possible.

A few genes that we found to contain very significant gaps in transposon coverage have been successfully deleted, and produce strains that grow normally under similar conditions to those used in our study [33], [34], [35], [36], [37], [38]. The reasons for these apparently contradictory results likely vary for each gene. It is possible that some of these genes are truly dispensable for *in vitro* growth, and the identified gaps in transposon coverage are either due to unappreciated transposon specificity, or selection against specific protein truncations. Conversely, it is possible that deletion mutants lacking these genes appear to grow normally because extragenic suppressor mutations have accumulated. This phenomenon is well-documented for mutants generated by homologous recombination [39], but is very unlikely to affect transposon-mapping studies. These differences highlight the advantages and limitations of different genetic approaches for defining essential genes.

In addition to this qualitative analysis, we also quantitatively compared mutant pools grown under different conditions to understand how Mtb metabolizes cholesterol. A number of recent studies have demonstrated that Mtb mutants lacking the capacity to acquire or degrade cholesterol are defective for growth in animal models of TB [13], [14], [17], [40], [41]. In order to define a discrete set of ORFs required for growth on this carbon source, we applied a continuous function cutoff to our comparative data, which placed equal importance on fold change in representation and statistical significance. More traditional one-dimensional cutoffs would have excluded genes that were only moderately underrepresented but exceptionally significant, which our analysis includes. Nonetheless, in order to avoid false positive predictions, we set a relatively stringent cutoff, and it is likely that even more than the 96 identified genes contribute to growth in cholesterol.

In addition to the dedicated cholesterol catabolic functions, we found that the use of this compound as a source of both cellular energy and biosynthetic carbon requires a variety of central metabolic pathways. Some of these requirements are likely due to the simple shift from a glycolytic substrate to one that relies heavily on β-oxidation. However, the precise pathways we identified appear specific to the mixture of metabolites derived from cholesterol. For example, gluconeogenesis under these conditions is likely to be initiated by the conversion of pyruvate to PEP via the action of pyruvate phosphate dikinase (PpdK), whereas this pathway relies exclusively on phosphenolpyruvate carboxykinase (PckA) during growth on acetate [42]. In other bacteria, PpdK-mediated gluconeogensis is favored during growth in the presence of pyruvate [43], [44]. As pyruvate is produced both as a direct product of sterol catabolism and through the activity of the methylcitrate cycle, we speculate that the relative abundance of cholesterol-derived pyruvate favors the PpdK-mediated pathway. These observations indicate that different gluconeogenic pathways may be preferentially used by Mtb depending on the relative abundance of precursor metabolites.

Cholesterol acquisition is predominantly required for bacterial persistence during the chronic stage of Mtb infection in mice and for growth in the cytokine-stimulated macrophages that characterize this stage of infection [14], [17]. While cholesterol is metabolized by the bacterium throughout infection [13], [41], we have shown that this ability is not required for the initial growth of the pathogen in acutely-infected animals or in the resting macrophages that model this early pre-immune period. A recent study demonstrating that cholesterol catabolism is not necessary for bacterial growth in acutely-infected guinea pigs or a macrophage cell line confirms these observations [45]. Thus, it appears that a mixture of carbon sources, including cholesterol, fuel the initial growth of the bacterium, and this sterol becomes a uniquely essential nutrient in chronically-infected animals. Based on these observations, we predict that the requirements for both the dedicated sterol catabolic enzymes, as well as the central metabolic pathways we have defined, are likely to change as infection proceeds.

Identifying the full complement of cellular functions necessary for cholesterol utilization has also revealed the scale of this nutritional adaptation during infection. When we compared these data to previous genome-wide screens for mutants attenuated in mouse models of infection [46] we found that a full ten percent of genes specifically required for bacterial growth *in vivo* are also required for the utilization of cholesterol *in vitro* (Table S3). These genes encode both dedicated sterol catabolic functions, as well as enzymes involved in central metabolism. Thus, while it is clear that Mtb must adapt to a variety of host-specific conditions to sustain a productive infection, our data suggest that a single nutritional change is responsible for a significant portion of this adaptation. These cholesterol catabolic functions, in conjunction with the hundreds of other genes that we found to be essential for bacterial viability, both expand and refine the repertoire of targets for new TB therapies.

Whole genome profiling techniques have proven to be useful tools for understanding complex pathways, such as those required for cholesterol utilization. Most of these approaches rely on determining the relative transcript or protein abundance. However, these strategies make a major assumption – that critical genes are tightly regulated in response to metabolic changes. While this may be true in many cases, it is often not. In order to avoid this assumption, we have directly identified the genes required for growth. While every approach has its own inherent strengths and weaknesses, the phenotypic profiling strategy described here is a powerful complementary tool for understanding bacterial physiology.

## Methods

### Mtb growth and selection

The transposon library was generated in the H37Rv background as previously described [46], and was comprised of approximately 10^5^ independent insertion events. 10^6^ colony forming units (cfu) of library were inoculated into 200 ml of minimal media (asparagine 0.5 g/L, KH_2_PO_4_ 1.0 g/L, Na_2_HPO_4_ 2.5 g/L ferric ammonium citrate 50 mg/L, MgSO_4_ ·7H_2_0 0.5 g/L, CaCl_2_ 0.5 g/L, ZnSO_4_ 0.1 mg/L), 25 µg/ml Kanamycin, 0.2% tyloxapol, 0.2% ethanol and either 0.1% glycerol or 0.01% cholesterol. Selections were carried out in duplicate for glycerol and triplicate for cholesterol. Libraries were grown in roller bottles at 37°C. Cultures were diluted as necessary to maintain the optical density at 600 nm below 0.2. The number of cell generations was monitored by cfu enumeration. Transposon mutants shown in Figure 3C were obtained from the Biodefense and Emerging Infections Research Resources Repository and are in the CDC1551 background.

### Genomic library preparation

Genomic DNA isolation, partial restriction digestion, ligation to asymmetric adapters, and transposon junction amplification were performed as described [5]. An additional nested PCR with the following oligonucleotides was used to incorporate Illumina attachment and sequencing sites AATGATACGGCGACCACCGAGATCTACACTCTTTCCCTACACGACGCTCTTCCGATCTCGGGGACTTATCAGCCAACC and CAAGCAGAAGACGGCATACGAGATCGGTCTCGGCATTCCTGCTGAACCGCTCTTCCGATCTGTCCAGTCTCGCAGATGATAAGG. Standard PCR (denaturation at 94°C for 30 seconds, annealing at 57.5°C for 30 seconds and extension at 72°C for 30 seconds) was performed for 9 cycles. Amplified fragments between 250–400 bp were purified and sequenced using either the primer: CCGGGGACTTATCAGCCAACC (complementary to the transposon inverted terminal repeat, ITR) or ACACTCTTTCCCTACACGACGCTCTTCCGATCT (complementary to the Illumina adapter) using an Illumina GA2 instrument. Illumina attachment and sequencing oligonucleotide sequences © 2006 Illumina, Inc.

### Sequence analysis

The sequencing reads that contained the Himar1 ITR sequence and the adjacent TA insertion site were identified in the raw fasta files and trimmed of the ITR sequence. The sequences were aligned to the *M. tuberculosis* H37Rv reference genome [47] using SOAPv1.11 alignment software [48] at default settings (2 mismatches allowed per read). A custom PERL script was used to extract the TA dinucleotide insertion site coordinates from the SOAP output file. For reads aligning to the plus strand of the genome, the genome coordinate at position 1 of the trimmed read was determined. For reads aligning to the minus strand, the genome coordinate at position 2 of the read was calculated to represent the TA coordinate position with respect to the plus strand. For each TA insertion site detected by alignment, the total number of reads and the strand orientation was determined. Sequence reads that aligned to more than one chromosomal position were randomly assigned to one of the positions. If less than 10% of the reads corresponding to an insertion site could be assigned to this single position, the TA site was removed from all further analyses. Less than 0.01% of TAs were excluded from any analysis. Insertion site coordinates were mapped to positions within protein coding genes annotated in RefSeq file NC_000962.ptt (from the National Center for Biotechnology Information: ftp://ftp.ncbi.nih.gov/).

### Essential gene analysis

Genes were scored for essentiality based on the length of the longest contiguous run of TA dinucleotide sites lacking observed insertions within the coding region. Read counts at each TA site were pooled from both strands and summed over all the sequencing runs for each library/growth condition (2 runs for growth on glycerol, 3 runs for growth on cholesterol), and sites with 5 or fewer observed reads were treated as non-insertions. The longest consecutive sequence of non-insertion sites, *k,* in each gene was identified. The expectation for the length of the longest run of TA sites lacking insertion in a gene with *n* TA sites is given by the following statistics for a sequence of Bernoulli trials with success probability θ:

µ = log_(1/θ)_(n(1−θ))+γ/ln(1/θ), σ = [π^2^/(6 ln^2^(1/θ)+1/12)]^−1/2^, where γ = 0.577... is the Euler-Mascheroni constant. In this case, θ represents the probability of insertions being observed at TA site in non-essential genes, which was conservatively estimated to be the fraction of TA sites containing insertions in the entire genome. To assess the statistical significance of the observed maximum run of sites without insertions, this was compared to the expected length of the longest run using the cumulative function of the Extreme Value Distribution to calculate a p-value: p(k;n,µ σ) = 1−exp(−exp(−(k−σ)σ), where µ and σ are the statistics of the expected distribution given above.

### Differential growth analysis

For a given protein coding gene, only detected insertion sites in the 5′ 5–80% of gene were considered. Samples were normalized such that the mean number of sequence reads per insertion site were equal. The relative representation of each mutant was determined by calculating the fold change (sequence reads/insertion in cholesterol divided by sequence reads/insertion in glycerol) for each gene. Statistical significance was determined by t-test. Each insertion site in each replicate sample was treated as a separate data point. The hyperbola used for defining genes specifically required for growth in cholesterol was defined by the formula, y = 3.8/x+0.7. Genes above this line are annotated as required for growth on cholesterol in Table S4.

## Supporting Information

Table S1 **Sequence reads detected per TA insertion site.**
(XLSX)

Table S2 **Analysis of essentiality** ***in vitro*** **.**
(XLSX)

Table S3 **Differential growth in glycerol and cholesterol media**
(XLSX)

Table S4 **Genes required for growth in cholesterol**
(XLSX)
